# The homeobox gene *DLX4* promotes generation of human induced pluripotent stem cells

**DOI:** 10.1038/srep07283

**Published:** 2014-12-04

**Authors:** Naritaka Tamaoki, Kazutoshi Takahashi, Hitomi Aoki, Kazuki Iida, Tomoko Kawaguchi, Daijirou Hatakeyama, Masatoshi Inden, Naoyuki Chosa, Akira Ishisaki, Takahiro Kunisada, Toshiyuki Shibata, Naoki Goshima, Shinya Yamanaka, Ken-ichi Tezuka

**Affiliations:** 1 Department of Oral and Maxillofacial Science, Gifu University Graduate School of Medicine, 1-1 Yanagido, Gifu City, Gifu 501-1194, Japan; 2 Center for iPS Cell Research and Application, Kyoto University, 53 Kawahara-cho, Shogoin, Sakyo-ku, Kyoto 606-8507, Japan; 3 Department of Tissue and Organ Development, Gifu University Graduate School of Medicine, 1-1 Yanagido, Gifu City, Gifu 501-1194, Japan; 4 Laboratory of Medical Therapeutics and Molecular Therapeutics, Gifu Pharmaceutical University, 1-25-4 Daigaku-Nishi, Gifu 501-1195, Japan; 5 Division of Cellular Biosignal Sciences, Department of Biochemistry, Iwate Medical University, 19-1 Uchimaru, Morioka, Iwate 020-8505, Japan; 6 Molecular Profiling Research Center for Drug Discovery, National Institute of Advanced Industrial Science and Technology, 2-4-7 Aomi, Koto-ku, Tokyo 135-0064, Japan

## Abstract

The reprogramming of somatic cells into induced pluripotent stem cells (iPSCs) by defined transcription factors has been a well-established technique and will provide an invaluable resource for regenerative medicine. However, the low reprogramming efficiency of human iPSC is still a limitation for clinical application. Here we showed that the reprogramming potential of human dental pulp cells (DPCs) obtained from immature teeth is much higher than those of mature teeth DPCs. Furthermore, immature teeth DPCs can be reprogrammed by OCT3/4 and SOX2, conversely these two factors are insufficient to convert mature teeth DPCs to pluripotent states. Using a gene expression profiles between these two DPC groups, we identified a new transcript factor, distal-less homeobox 4 (DLX4), which was highly expressed in immature teeth DPCs and significantly promoted human iPSC generation in combination with OCT3/4, SOX2, and KLF4. We further show that activation of TGF-β signaling suppresses the expression of DLX4 in DPCs and impairs the iPSC generation of DPCs. Our findings indicate that DLX4 can functionally replace c-MYC and supports efficient reprogramming of immature teeth DPCs.

Previous reports showed that induced pluripotent stem cells (iPSCs) can be generated from various types of somatic cells using a combination of transcription factors, such as OCT3/4, SOX2, KLF4, c-MYC, NANOG, and LIN28^1^,^2^,^3^,^4^,^5^,^6^,^7^,^8^,^9^,^10^,^11^,^12^,^13^,^14^. Although these reports indicated that iPSCs can be induced from many types of somatic cells, the backgrounds of the donor cells, such as gene expression patterns and the epigenetic state, have a significant impact on the reprogramming efficiency, kinetics, and the transcription factors required for the induction^3^,^5^,^7^,^13^,^14^,^15^,^16^. Because reprogramming of differentiated cells involves gradual changes in gene expression patterns, cell morphology, and epigenetic state towards the embryonic state^17^,^18^,^19^,^20^,^21^, it has been assumed that stem cells and progenitor cells are more amenable to reprogramming than that of more differentiated cells^5^,^12^,^16^,^22^,^23^. For example, mouse and human neural stem cells can be reprogrammed with the single transcription factor OCT3/4^16^,^24^. Similarly, Eminli et al. reported that hematopoietic stem and progenitor cells gave rise to iPSCs up to 300 times more efficiently than terminally differentiated B and T cells did, suggesting that the differentiation state of the starting cells affects reprogramming efficiency^23^. However, these stem/progenitor cells are generally inaccessible, require complicated procedures to obtain, and are difficult to propagate *in vitro*.

Human dental pulp cells (DPCs) are obtained easily from adult teeth discarded as medical waste and contain abundant stem like cells^25^,^26^,^27^,^28^,^29^. In addition, DPCs can be expanded in a simple culture condition and show continuous growth for up to 30 passages^28^. In a previous study, we established more than 150 DPC lines isolated from extracted wisdom teeth and evaluated the potential of DPCs for iPS cell banking^14^,^28^. The donor teeth of these DPC lines were categorized into three developmental stages: crown completed (CC), root forming (RF), and root completed (RC). We randomly selected six lines isolated from various developmental stages to induce iPSCs and found that five DPC lines showed higher reprogramming efficiency than human dermal fibroblasts (HDFs). In this study, it remained unclear why DP75, isolated from the oldest patient in the RC stage, showed remarkably low reprogramming efficiency similar to HDFs.

Here, we focused on the relationship between the developmental stages of DPCs and reprogramming efficiency. We found that DPCs in the mature (RC) stage were reprogrammed less efficiently than DPCs in the immature (CC and RF) stages.

## Results

### DPCs isolated from immature teeth can be reprogrammed into iPSCs using two factors

**Figure 1:**
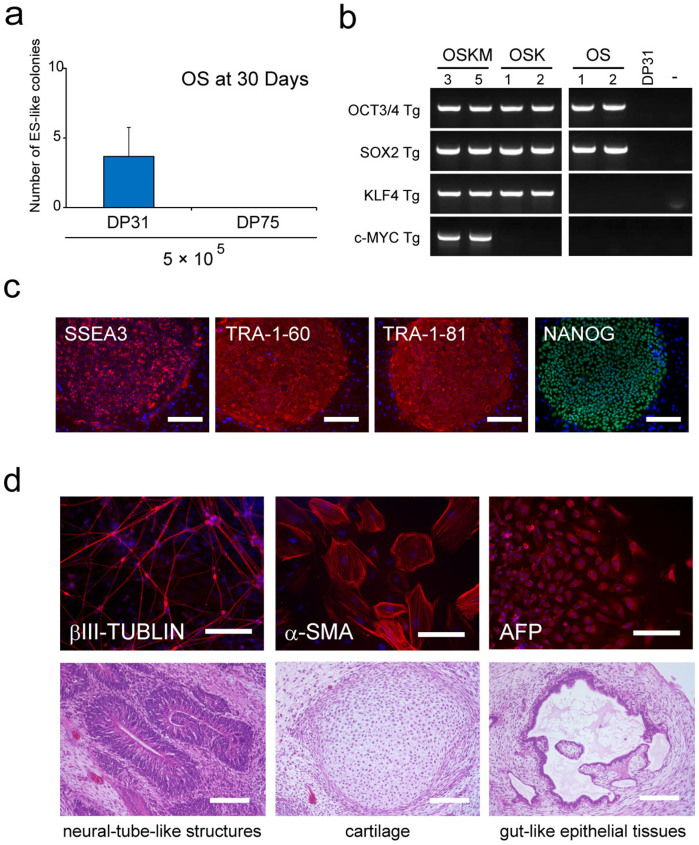
DP31 cells can be reprogrammed to iPSCs using OCT3/4 and SOX2. (a) We obtained a few ES-cell-like colonies from 5 × 10^5^ DP31 cells transduced with OCT3/4 and SOX2 (OS), but we could not obtain any ES-cell-like colonies from DP75 cells. Human ES-cell-like colonies were counted at 30 days post infection. Error bars indicate ± S.D. (n = 3). (b) Genomic PCR using transgene-specific primers, with DP31 cells as a negative control, confirmed the insertion of only two transgenes in iPSCs derived from DP31 cells transduced with OS (iPS-DP31-OS) by PCR. The numbers denote different iPSC lines. We cropped the gels and blots for clarifying our presentation. The gels have been run under the same experimental conditions. (c) iPS-DP31-OS cells expressed pluripotency markers including SSEA3, TRA-1-60, TRA-1-81, and NANOG. Scale bar = 100 μm. (d) Pluripotency of iPS-DP31-OS cells was confirmed by EB-mediated differentiation and teratoma formation assay. Immunofluorescence staining showed that EB structures derived from iPS-DP31-OS cells expressed markers characteristic of the three germ layers including βIII-tubulin (ectoderm), α-smooth-muscle actin (mesoderm), and α-fetoprotein (endoderm). Nuclei were stained with Hoechst 33342. Scale bar = 100 μm. Hematoxylin- and eosin-stained sections of teratomas generated from iPS-DP31-OS cells are shown in the lower panels. The teratomas contained various tissues of all three germ layers, such as neural-tube-like structures (ectoderm), cartilage (mesoderm), and gut-like epithelial tissue (endoderm). Abbreviations: AFP, alpha-fetoprotein; α-SMA, alpha smooth muscle actin. Scale bar = 100 μm.

First, we attempted to generate iPSCs from DP31 cells, a cell line isolated from the immature (CC) stage of teeth, using as few transcription factors as possible. We found that only two factors, OCT3/4 and SOX2 (OS), are required to induce ES-cell-like colonies repeatedly, albeit with very low frequency compared to the combination of OCT3/4, SOX2, and KLF4 (OSK) (Fig. 1a). Similarly, we obtained iPSCs from other immature tooth DPCs, such as DP1 (CC stage) and DP87 (RF stage), with only two factors (data not shown). Genomic PCR was employed to demonstrate that OS-induced iPSCs derived from DP31 cells (iPS-DP31-OS) contained only OCT3/4 and SOX2 transgenes (Fig. 1b). We also confirmed that iPS-DP31-OS cells could be expanded easily and expressed human specific pluripotency markers (Fig. 1c). These cells differentiated into all the three germ layers *in vitro* and *in vivo* (Fig. 1d). However, we did not obtain any ES-cell-like colonies from DP75, a line obtained from the mature (RC) stage of the tooth, using the OS cocktail (Fig. 1a). These results compelled us to further examine the maturation stage dependency of DPC reprogramming.

### Statistical comparison of reprogramming potency between immature and mature tooth DPCs

**Figure 2:**
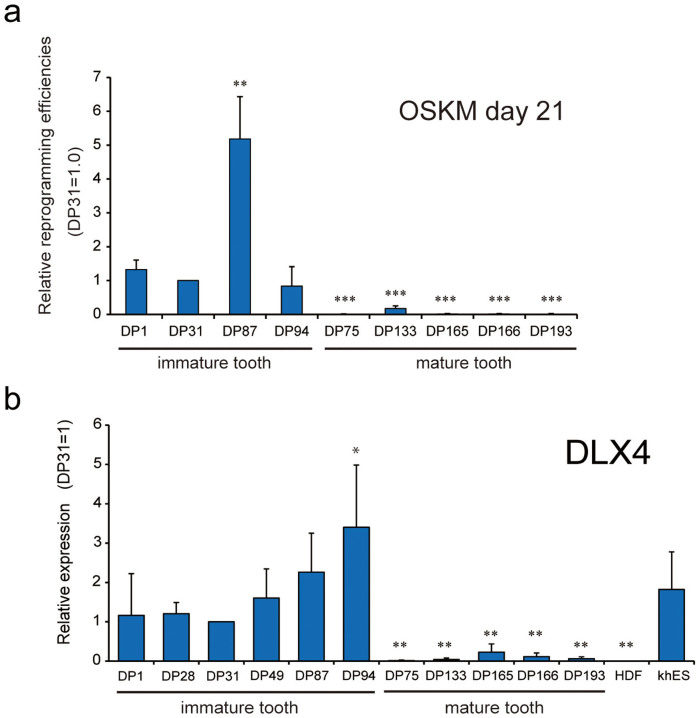
DPCs from immature teeth were more amenable to reprogramming than DPCs from mature teeth and showed high expression levels of endogenous DLX4. (a) Nine DPC lines were selected for evaluation of reprogramming potency. The four Yamanaka factors (OSKM) were introduced by a retroviral system, and the induced human ES-cell-like colonies were counted at 21 days post infection. The number of human ES-cell-like colonies obtained from each DPC line was normalized against that obtained from DP31 cells. Error bars indicate ± S.D. (n = 3). Asterisks indicate statistical significance: *P < 0.05, ** P < 0.01, and *** P < 0.001 compared to DP31 values. (b) The transcript levels of endogenous DLX4 were quantified using real-time RT-PCR. DPCs from immature teeth showed significantly higher levels of endogenous DLX4 than DPCs from mature teeth. Moreover, the transcript levels of DLX4 in all immature teeth DPCs were similar to those of human ES cells (hKES). GAPDH was used as an internal control. Error bars indicate ± S.D. (n = 3). Asterisks indicate statistical significance: *P < 0.05, ** P < 0.01, and *** P < 0.001 compared to values from DP31 cells.

To confirm that the reprogramming potency of DPC lines was dependent on the developmental stage but not on simple individual differences of donor teeth, we selected an additional five DPC lines (nine lines in total) isolated from various developmental stages (Supplementary Fig. S1) and introduced Yamanaka's four factors (OCT3/4, SOX2, KLF4, c-MYC; OSKM) into them using a retroviral gene-delivery system. Human ES-cell-like colonies were morphologically identified and counted at 21 days post-infection as previously described^1^,^14^. The number of human ES-cell-like colonies obtained from each DPC line was normalized to that of DP31 cells. All DPC lines isolated from the immature teeth (CC and RF stages) group showed significantly higher reprogramming efficiency than the lines isolated from mature (RC stage) teeth (Fig. 2a).

### Identification of differentially expressed genes in DPCs isolated from immature and mature teeth

The results of the reprogramming experiments prompted us to compare the gene expression profiles of DPCs from mature and immature teeth. To avoid masking of target genes by sex-related genes, we chose eight DPC lines isolated from male donors (four lines from individuals with immature teeth and four lines from individuals with mature teeth) (Supplementary Fig. S1). Among the 28 genes significantly up-regulated by more than five-fold in immature teeth DPCs (Supplementary Table S1), we focused on the homeobox gene *Dlx4*, the expression of which was up-regulated 35-fold in immature teeth DPCs on average and was thus the most differentially expressed transcription factor.

We performed a real-time PCR assay to evaluate the transcript levels of DLX4 in each DPC line. As expected, the transcript levels of DLX4 in DPCs from immature teeth were much higher than those of mature teeth DPCs and HDFs (Fig. 2b). Moreover, the transcript levels of DLX4 in all immature teeth DPCs were similar to those of human ES cells (hKES).

### Ectopic expression of *DLX4* promotes human iPSC generation

**Figure 3:**
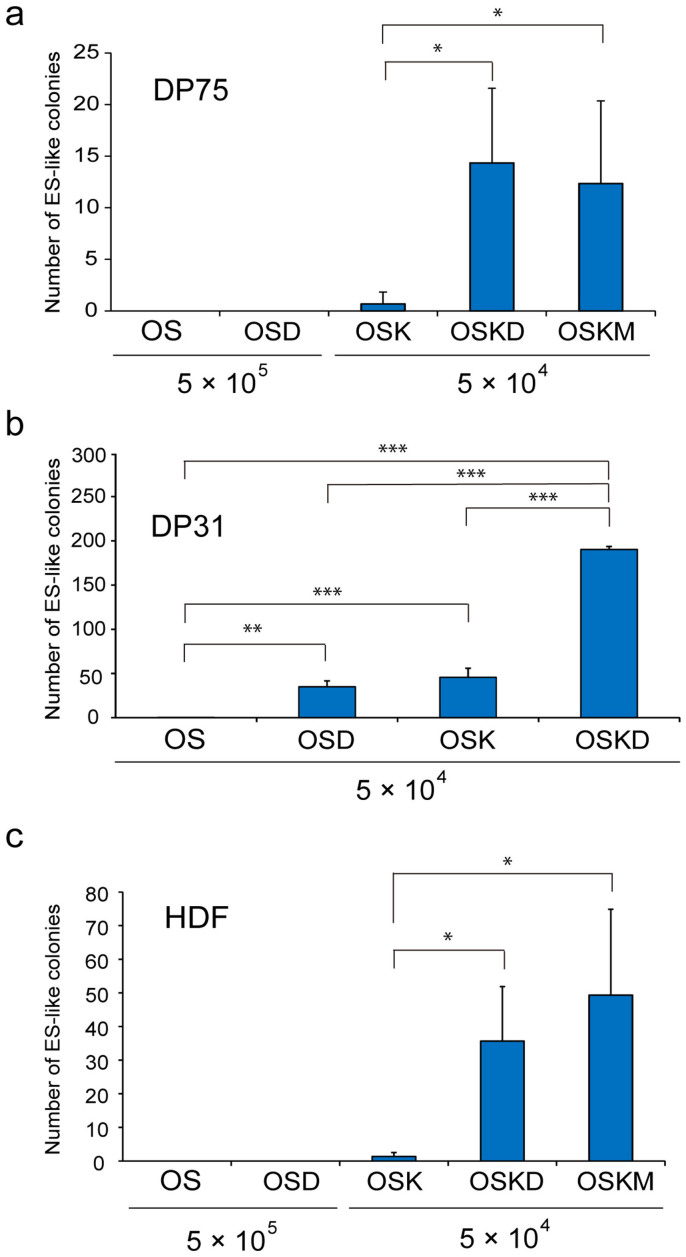
Ectopic expression of DLX4 promoted human iPSC generation. (a-c) Numbers of human ES-cell-like colonies obtained from DPCs and HDFs. Human ES-cell-like colonies isolated from 5 × 10^5^ or 5 × 10^4^ infected cells were counted at 30 days post infection. Error bars indicate ± S.D. (n = 3). Asterisks indicate statistical significance; *P < 0.05, **P < 0.01, and ***P < 0.001.

We next studied whether ectopic expression of DLX4 could increase the reprogramming efficiency of DPCs. Because we could not obtain any ES-cell-like colonies from mature teeth DPCs using the OS combination, we tried co-expression of DLX4 with OS (OSD) to convert DP75 into pluripotent cells. Contrary to our expectation, we could not obtain any ES-cell-like colonies from DP75 using the OSD cocktail (Fig. 3a). However, we observed a synergistic increase in the number of ES-cell-like colonies when both DLX4 and KLF4 were co-introduced with OS (OSKD) (~0.03%). The number of ES-cell-like colonies obtained from DP75 with OSKD was similar to the number obtained using OSKM (Fig. 3a). Next, we addressed whether ectopic expression of DLX4 could produce a similar effect during the reprogramming of DP31 cells obtained from immature teeth. As observed in DP75 cells, ectopic expression of DLX4 enhanced the reprogramming efficiency of DP31 cells (Fig. 3b). Although we could not obtain any ES-like colonies from 5 × 10^4^ introduced DP31 cells with OS combination, we could obtain about 30 ES-like colonies constantly by using OSD combination. The OSD combination increased the reprogramming efficiency of DP31 (~0.07%) to the same level provided by OSK (Fig. 3b). As observed in DPCs, the OSKD cocktail increased the reprogramming efficiency in HDF cells to the same level observed using OSKM, although we did not obtain any ES-cell-like colonies with OSD alone, as observed for DP75 cells (Fig. 3c). Our results indicate that ectopic expression of DLX4 enhances reprogramming efficiency in combination with OCT3/4 and SOX2, similarly to KLF4. Notably, we found that use of the OSKD combination not only enhanced reprogramming, but also prevented the generation of non-ES-cell-like colonies to a greater extent than was observed with OSKM (Supplementary Fig. S2).

To determine whether enhanced reprogramming efficiency by DLX4 overexpression could result in stable establishment of iPSC lines, we picked nine ES-cell-like colonies derived from DP31 with OSD (termed iPS-DP31-OSD) for further examination. Among them, eight colonies were successfully expanded. Immunostaining confirmed the expression of pluripotent markers, including OCT3/4, SSEA4, TRA-1-60, and TRA-1-81, at levels similar to those in human ES cells (Supplementary Fig. S3a). Using real-time PCR analysis, we found that transcript levels of the pluripotency markers OCT3/4 (endogenous), NANOG, and REX1 were markedly increased in iPS-DP31-OSD lines relative to the parental cells and were similar to the transcript levels in human ES cells (Supplementary Fig. S3b). Differentiation potential towards the three germ layers was also confirmed *in vitro* and *in vivo* (Supplementary Fig. S3c).

### Activation of TGF-β signal suppresses the expression level of DLX4 in DPCs and impairs iPSC generation from DPCs

**Figure 4:**
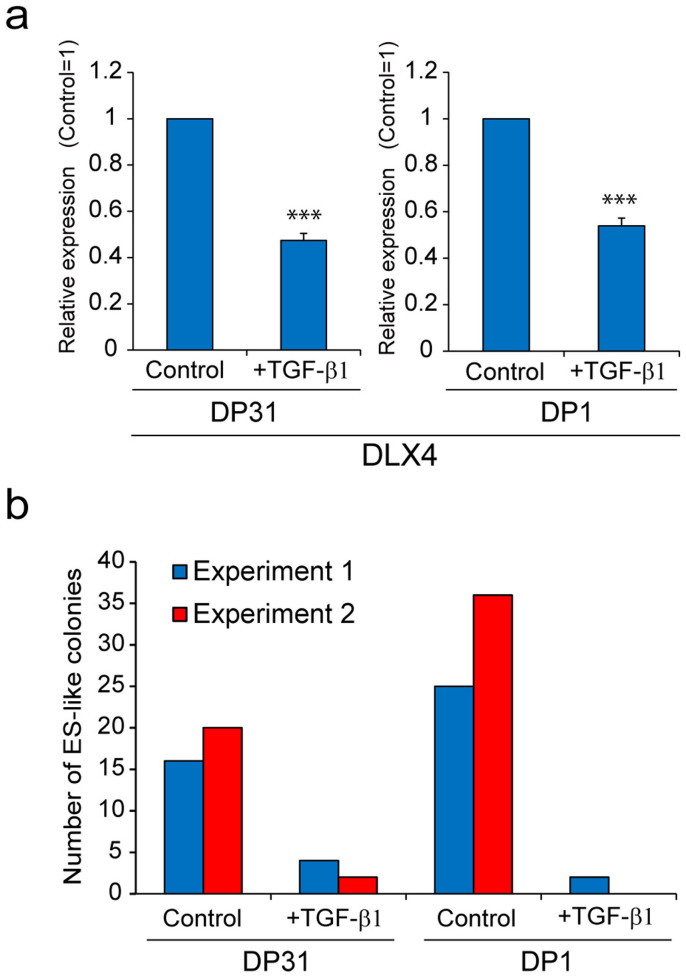
Activation of TGF-β signal suppressed the expression level of DLX4 in DPCs and impaired the generation of iPSCs from DPCs. (a) DP1 and DP31 cells were cultured in DPC culture medium with TGF-β1 (5 ng/mL) or with acetic acid (as a control) for 48 h. Real-time PCR analysis showed that after activation of TGF-β signal, the transcript levels of DLX4 were down-regulated in both DP1 and DP31 cells. GAPDH was used as an internal control. Error bars indicate ± S.D. (n = 3). Asterisks indicate statistical significance; ***P < 0.001 (b) Human ES-cell-like colonies isolated from 5 × 10^4^ infected cells (OSKM) were counted at 21 days post infection. Numbers of human ES-cell-like colonies obtained from DP1 and DP31 were dramatically reduced when TGF-β1 (5 ng/mL) was added during the reprogramming process.

Previous reports showed that MET (mesenchymal-to-epithelial transition) is a crucial early process for direct reprogramming of mouse fibroblasts to pluripotent status and that ectopic expression of KLF4 potently induced CDH1 (E-cadherin) mRNA to promote MET^19^,^20^,^30^. Similarly, KLF4 alone robustly induced CDH1 in DP31 cells as previously reported with mouse fibroblasts^20^,^30^ (Supplementary Fig. S4a). Interestingly, ectopic expression of DLX4 also up-regulated CDH1. However, the potency of CDH1 mRNA up-regulation appeared to be lower than that of KLF4. Recent reports showed that using an inhibitor of TGF-β signaling, which promotes EMT (epithelial to mesenchymal transition), could enhance iPSC generation from mouse and human fibroblasts^20^,^31^,^32^,^33^. We then attempted to address whether TGF-β signaling could affect nuclear reprogramming of DPCs. Interestingly, the expression level of endogenous DLX4 in DPCs was suppressed by TGF-β signaling (Fig. 4a). Moreover, addition of TGF-β1 dramatically reduced the number of iPSC colonies obtained from DP1 and DP31 cells (Fig. 4b). Our results indicated that activation of TGF-β signaling suppresses the expression of DLX4 in DPCs and impairs the generation of iPSC from DPCs.

Since TGF-β signal also plays a crucial role in regulation of the cell cycle^34^,^35^ and acceleration of cell cycle is a crucial process in early stage of reprogramming^21^. Next, we addressed whether enforced expression of DLX4 could affect the cell cycle in DPCs. Firstly, we tested whether enforced expression of DLX4 can activate endogenous expression of c-MYC in DPCs, because of its potential to replace c-MYC during nuclear reprogramming. Contrary to our expectations, we could not observe the up-regulation of c-MYC in DLX4 induced DP31 cells (Supplementary Fig. S4b). Similar to this, we also could not observe the down-regulation of CDK inhibitors by ectopic expression of DLX4, even with the combination of OSK (Supplementary Fig. S4b). Our results indicated that DLX4 does not accelerate cell growth in the early stage of reprogramming to promote iPSC generation.

## Discussion

In this study, we have identified a new transcription factor, DLX4, also known as DLX7 or BP1, which promotes human iPSC generation in combination with the classical Yamanaka factors. In mammals, the *Dlx* gene family consists of six members that are organized into three closely linked pairs: *Dlx1*/*Dlx2*, *Dlx5*/*Dlx6*, and *Dlx3*/*Dlx4*^36^,^37^,^38^. These *Dlx* genes play crucial roles in early development^39^,^40^,^41^,^42^ and are known to be expressed in cranial neural crest cells and later in the craniofacial mesenchyme^43^. In this study, we found that DPCs isolated from developing teeth in the CC or RF stage show high expression of DLX4. Some reports have demonstrated that *Dlx* genes are associated with mammalian tooth formation^39^,^44^, and indicated that DLX4 is closely related to abnormal human tooth formation. For example, a mutation of DLX3, which is the partner of DLX4, is associated with tricho-dento-osseous (TDO) syndrome and disrupts odontoblast polarization and dentin formation^45^. Therefore, it is conceivable that DLX4 is highly expressed in DPCs isolated from third molar teeth, which are still under development even after birth.

Previously, Maekawa et al. attempted to identify new transcription factors with the ability to replace Klf4 during nuclear reprogramming of mouse skin fibroblasts from their library of 1,437 human transcription factors^46^. DLX4 was in their library, but was not included in the final screening. We could not obtain any ES-cell-like colonies from HDFs using the OSD cocktail. Conversely, we found that DLX4 promoted iPSC generation in HDFs only in the presence of KLF4. Therefore, it is possible that ectopic expression of DLX4 alone did not improve reprogramming efficiency in HDFs. It was thus excluded from the candidate list in the previous study.

Here we showed that the OS cocktail converted DP31 into a pluripotent state, although cells isolated from mature teeth (DP75) failed to be reprogrammed. This result indicates that DPCs isolated from immature teeth have unique properties. In this study, we have revealed that ectopic expression of DLX4 significantly promotes human iPSC generation; accordingly high expression levels of DLX4 in immature teeth DPCs are helpful for their effective reprogramming. Our results suggest that DLX4 plays a crucial role during reprogramming of DPCs and controls their reprogramming efficiency. However, the regulation of reprograming efficiency by DLX4 does not fully explain the unique properties of immature teeth DPCs. For example, the OSD cocktail was still insufficient to convert DP75 to a pluripotent state, conversely it up-regulated the reprogramming efficiency of DP31 to the same level provided by OSK. Moreover, DP87 shows substantially higher reprogramming efficiency but the expression level of DLX4 is average compared to other immature teeth DPC lines. This indicated that the measured endogenous DLX4 expression level determined by real-time PCR did not completely correlate with the reprogramming efficiency of DPCs and endogenous DLX4 expression may not be the sole factor responsible for the reprogramming efficiency to DPCs. A plausible explanation is that DLX4 may function together with some partner gene(s) to promote reprogramming, so the expression level of this (these) gene(s) may also affect the reprogramming efficiency of DPCs. Further screening of the genes listed in Supplemental Table S1 may lead to the discovery of additional factors that work together with DLX4.

An important remaining question is how DLX4 acts on a molecular level to promote reprogramming process in DPCs. Trinh et al. showed that DLX4 induces c-MYC and inhibits TGF-β mediated induction of p15^Ink4B^ and p21^WAF1/Cip1^ expression by blocking the TGF-β/Smad signaling pathway in normal and malignant epithelial cell lines^47^. Blockage of TGF-β signaling leads to promotion of MET, which is critical initiating event during the derivation of iPSCs from mesenchymal cells^19^,^20^. In this study we also observed that enforced expression of DLX4 in DPCs caused modest up-regulation of CDH1, however compared to KLF4, the potential to induce CDH1 is very mild. It was initially speculated that DLX4 may promote reprogramming process by activating the endogenous expression of c-MYC, because we found that DLX4 can restore the reprogramming efficiencies of DPCs and HDFs in the absence of c-MYC. The close correlation between DLX4 and c-MYC also has been shown in other cell types, such as hematopoietic cells^48^,^49^. Contrary to our expectation, we could not observe the up-regulation of c-MYC after induction of DLX4 in DPCs. Similar to c-MYC, enforced expression of DLX4 also did not affect the transcript levels of CDK inhibitors, such as p21^WAF1/Cip1^, p15^Ink4B^, and p16^Ink4A^, which have been demonstrated as roadblocks against reprogramming^18^,^50^,^51^,^52^,^53^. On the other hand, we did observe that TGF- β suppressed the expression of DLX4 in DPCs and impaired iPSC generation. Although the TGF-β/activin signaling pathway is important for the maintenance of human embryonic stem cells^54^,^55^, our results suggested that the TGF-β signaling associated with reduction of DLX4 is one of the roadblocks for effective reprogramming of human somatic cells. Since homeobox genes often function in a context dependent and lineage specific manner, the functions of DLX4 may be depended on the cell types. Although we have tested two mesenchymal linages of somatic cells whether DLX4 can promote reprogramming, it is desirable to test another cell types, such as epithelial and hematopoietic cells.

In this study we used conventional retroviral system to induce iPSCs and our process contains splitting step at 6 days post infection. So we cannot formally exclude the possibility that we might count the ES-like colonies derived from the same clones and this could result in overestimation of reprogramming efficiencies. But as mentioned above, it was unlikely that DLX4 induced proliferation in DPCs before the splitting step and accordingly the number of ES-like colonies obtained from using DLX4 was increased.

Here we show that DPCs isolated from immature teeth are more easily reprogrammed into iPSCs than DPCs derived from adult teeth and identify *DLX4* as one of the candidate genes that is differentially expressed during the developmental stage. To our knowledge, this is the first report of a new transcript factor that enhances iPSC generation based on a comparative analysis of gene expression profiles using human cell resources. Our findings also provide evidence that our DPC collection obtained from many donors is a promising resource for both basic and clinical studies toward setting up a human iPS cell bank.

## Methods

### Cell culture

The isolation of DPCs was described in our previous report. DPCs were cultured in MSCGM (Lonza) and HDFs were cultured in DMEM with 10% FBS containing penicillin/streptomycin. The human ES cell line (KhES01) was obtained from Kyoto University and cultured on mitomycin C-treated SNL feeder cells in Primate ES cell medium (ReproCELL) supplemented with 4 ng/mL bFGF (WAKO). Human iPSCs were generated and maintained in the human ES cell culture condition.

### Virus production and iPSC generation

We first introduced the mouse receptor for retroviruses (Slc7a1) into DPCs and HDFs using a lentiviral system according to our previous report^1^,^14^. Complementary DNA for human *OCT3/4, SOX2, KLF4*, and *c-MYC* was cloned into the retroviral pMXs vector, and *DLX4* was transferred to the pMXs-GW retroviral expression vector using the Gateway LR reaction. These pMXs-based retroviral vectors were separately transfected into Plat-E cells (ecotropic retrovirus packaging cells) using Fugene 6 (Roche). We also used a pMX retroviral vector encoding enhanced green fluorescent protein (*EGFP*) to monitor transduction efficiency by fluorescence-activated cell analysis (FACSAria, Beckton Dickinson). Twenty-four hours after transfection, the medium was replaced with fresh medium. After 24 hours, the viral supernatant was collected and mixed with an equal amount of each factor to infect HDFs/Slc7a1 and DPCs/Slc7a1 in medium supplemented with 4 μg/mL polybrene (Nacalai Tesque). Six days after retroviral infection, HDFs/Slc7a1 and DPCs/Slc7a1 were harvested by trypsinization and replated at 5 × 10^4^ or 5 × 10^5^ cells per 100-mm dish on mitomycin C-treated SNL feeder cells. The next day, the medium was replaced with primate ES cell medium supplemented with 4 ng/mL bFGF. Recombinant human TGF-β1 (reconstituted with 10 mM citric acid containing 0.1% BSA) was obtained from Pepro Tech. To evaluate the effect of activating TGF-β signal during reprogramming, we added TGF-β1 (5 ng/mL) into culture medium from day 1 to day 21 after retroviral infection, and counted the human ES-cell-like colonies obtained from 5 × 10^4^ infected DPCs at day 21.

### Assessment of reprogramming efficiency of DPC lines

To determine the reprogramming potential of DPCs isolated from various developmental stages, we introduced the OSKM transcription factors into nine DPC lines using a retroviral system. The generation of iPSCs was performed according to our general protocol. Since we found that the transduction efficiencies of DPCs are at similar levels in their early passages, we used DPCs within ten passages to induce iPSCs. We counted the human ES-cell-like colonies obtained from 5 × 10^4^ infected DPCs at 21 days after infection. The assays were conducted three times. The number of the human ES-cell-like colonies obtained from each DPC line was normalized to the number obtained from DP31. The relative reprogramming efficiency of DP31 was set as 1.0.

### Real-time PCR

Total RNA was isolated from the cells using an RNeasy Plus Mini Kit (Qiagen). Total RNA (500 ng) was used for the reverse transcription reaction with Rever Tra Ace-α (Toyobo) according to the manufacturer's instructions. For real-time PCR analysis, PCR amplification of cDNA was performed by using SYBR Premix Ex Taq (Takara) and analyzed using a Thermal Cycler Dice Real-Time System (Takara). Primer sequences are listed in Supplementary Table S2.

### Immunofluorescence staining

The cells were fixed with 4% paraformaldehyde, and treated with PBS containing 5% normal goat or donkey serum, 1% BSA, and 0.2% TritonX-100. The following antibodies were used: SSEA3 (1:10), TRA-1-81 (1:50), TRA-1-60 (1:50) (these antibodies were used at Kyoto University and were kind gifts from Dr. Peter W. Andrews), anti-SSEA4, anti-TRA-1-81, anti-TRA-1-60 (1:500, all contained in the ES Cell Characterization Kit from Merck Millipore; these antibodies were used at Gifu University), anti-NANOG (1:20, R&D Systems), anti-OCT3/4 (1:1000, Santa Cruz Biotechnology), anti-βIII-tubulin (1:200, Cell Signal Technology), anti-βIII-tubulin (1:2000, Covance), anti-α-SMA (1:500, DAKO), and anti-AFP (1:100, R&D). Nuclei were stained using 1 μg/mL Hoechst33342 (Life Technologies).

### EB formation and differentiation assay

For EB formation, we harvested iPSCs by treating them with collagenase IV. The clumps of cells were transferred to poly (2-hydroxyethyl methacrylate)-coated dishes containing DMEM/F12 supplemented with 20% knockout serum replacement (Life Technologies), 2 mM L-glutamine, 0.1 M non-essential amino acids, 0.1 M 2-mercaptoethanol (Life Technologies), and 0.5% penicillin/streptomycin. The medium was changed every other day. After eight days as floating cultures, EBs were transferred to gelatin-coated plates and cultured in the same medium for another eight days. Spontaneous germ-layer differentiation of EBs was assayed using markers for endoderm (AFP), mesoderm (α-SMA), and ectoderm (βIII-tubulin).

### Teratoma formation

For teratoma formation, 3 × 10^5^ iPSCs were injected into the testes of a SCID mouse using a Hamilton syringe. Nine weeks after injection, tumors were dissected and fixed with PBS containing 4% paraformaldehyde. After paraffin embedding, the tissue was sectioned and stained with hematoxylin and eosin.

### DNA microarray

Total RNA was prepared using the RNeasy Midi Kit (Qiagen). RNA (250 ng) was converted to cDNA, amplified, and labeled with Cy3 using the Quick Amp Labeling Kit (Agilent). Labeled DNA was hybridized to a Whole Human Genome Microarray Kit, 4 × 44 K (Agilent) according to the manufacturer's protocol. After hybridization, the arrays were washed consecutively using a Gene Expression Wash Pack (Agilent) and scanned using the G2505C Microarray Scanner System (Agilent). The data were normalized and analyzed by using GeneSpringGX11 (Agilent). The level of gene expression was determined as the average difference, and we selected genes with at least a five-fold difference in expression between DPCs from immature teeth and those from mature teeth. The microarray data was submitted to the NCBI GEO database under the accession number (GSE52853).

### Statistical analysis

The differences in mean values were assessed by using unpaired Student's t-test, with P < 0.05 considered statistically significant. Error bars indicate ± SD (n = 3).

## Author Contributions

N.T. performed and designed most of the experiments, analyzed the data, and wrote the manuscript. K.T. performed and designed the experiments for OS-induced iPSCs. H.A. and M.I. performed the characterization of iPSCs. K.I. and D.H. generated iPSCs from DPCs. T.K. performed the computer analyses of the DNA microarray data. N.C., A.I., T.K. and T.S. analyzed all data in this study. N.G. provided the retroviral expression clones. S.Y. supervised this study. K.T. conceived and supervised this study, designed the experiments, analyzed the data, and wrote and approved the manuscript.

## Additional Information

**Accession codes:** The microarray data were submitted to the NCBI GEO database under the accession number (GSE52853).

## Supplementary Material

Supplementary Information Supplementary Information
