# Supplementary material for: The homeobox gene DLX4 promotes generation of human induced pluripotent stem cells
Source: Sci Rep. 2014 Dec 4;4:7283. doi: 10.1038/srep07283 (PMC4255186; doi:10.1038/srep07283)
Supplement: Supplementary Information [file srep07283-s1.pdf]

(Supplementary Information)

The homeobox gene *DLX4* promotes generation of human induced pluripotent stem cells

Naritaka Tamaoki, Kazutoshi Takahashi, Hitomi Aoki, Kazuki Iida, Tomoko Kawaguchi, Daijirou Hatakeyama, Masatoshi Inden, Naoyuki Chosa, Akira Ishisaki, Takahiro Kunisada, Toshiyuki Shibata, Naoki Goshima, Shinya Yamanaka, and Ken-ichi Tezuka*

*corresponding author
Ken-ichi Tezuka: tezuka@gifu-u.ac.jp

Supplementary Figure S1 DPC lines used in this study. (a) Conventional X-ray photograph showing wisdom teeth (arrowheads) at various developmental stages: crown-completed, root-forming, and root-completed stages. (b) Various assays performed with each DPC line are indicated by "√". Stage indicates the developmental stages of teeth isolated from patients to establish DPC lines. The CC and RF stages are categorized to the immature tooth DPC group, and the RC stage is categorized to the mature tooth group. Abbreviations: CC, crown-completed stage; RF, root-forming stage; RC, root-completed stage.

Supplementary Figure S2 DLX4 specifically promoted the generation of ES-cell-like colonies. We counted the ES-cell-like colonies and total colonies obtained from 5×10^4 infected DP1 cells at 21 days post infection. Although the number of total colonies obtained from OSKM-induced DP1 cells was much larger than that from OSKD-induced cells, the numbers of ES-cell-like colonies were almost equal. Error bars indicate ± SD (n = 3). Asterisks indicate statistical significance: $*P < 0.05$ Abbreviations: Total; total colonies, iPS; iPSC colonies.

Supplementary Figure S3 Characterization of iPSCs isolated from DP31 cells

transduced with OCT3/4, SOX2, and DLX4 (OSD). (a) Immunofluorescence staining showed that iPS-DP31-OSD cells expressed pluripotency markers including OCT3/4, SSEA4, TRA-1-61, and TRA-1-80. Nuclei were stained with Hoechst 33342. Scale bar = 100 μm. (b) Real-time PCR analysis for pluripotency markers including NANOG, REX1, and OCT3/4 (endogenous) expressed in iPSCs isolated from DP31 cells transduced with OSD and OSKD. All iPSC clones analyzed expressed these genes at levels comparable to those of human ES cells (khES). In contrast, non-reprogrammed DP31 cells showed quite low expression of these genes. GAPDH was used as an internal control. (c) Embryoid body-mediated differentiation of iPS-DP31-OSD cells. Immunofluorescence staining showed that iPS-DP31-OSD cells differentiated into cells expressing markers characteristic of all three germ layers, including AFP (endoderm), α-SMA (mesoderm), and βIII-tubulin (ectoderm). Nuclei were stained with Hoechst 33342. Scale bar = 100 μm. (d) Hematoxylin and eosin-stained sections of teratomas generated from iPS-DP31-OSD cells. The teratomas contained various tissues of all three germ layers, such as gut-like epithelial tissue (endoderm), cartilage (mesoderm), and pigment epithelia (ectoderm). Scale bar = 100 μm. Abbreviations: AFP; alpha-fetoprotein, α-SMA; α-smooth muscle actin.

Supplementary Figure S4 Real-time PCR assay for the indicated genes at day 6 after induction of exogenous factors. (a,b) After transducing various combination of reprogramming factors into DP31 cells using a retroviral system, we cultured the cells in DPC culture medium for six days and analyzed the transcriptional alterations of indicated genes. GAPDH was used as an internal control. Error bars indicate ± S.D. ($n = 3$). Asterisks indicate statistical significance: ∗$P < 0.05$, and ∗∗∗ $P < 0.001$ compared to values of GFP.

Supplementary Table S1. Genes up-regulated more than 5-fold in immature teeth DPCs (vs. mature teeth DPCs).

Ratio	Gene Symbol	Gene ID	Official Full Name
38.1	*CD24*	100133941	CD24 signal transducer
35.1	*DLX4*	1748	distal-less homeobox 4, transcript variant 1
32.1	*NPTX2*	4885	neuronal pentraxin II,
26.2	*FGF13*	2258	fibroblast growth factor 13, transcript variant 1
21.7	*CPE*	1363	carboxypeptidase E
20.1	*SOX11*	6664	SRY (sex determining region Y)-box 11
16.2	*RBP1*	5947	retinol binding protein 1, cellular, transcript variant 1
9.89	*SFRP2*	6423	secreted frizzled-related protein 2
9.09	*TMSB15A*	11013	thymosin beta 15a
8.98	*FAT3*	120114	FAT tumor suppressor homolog 3 (Drosophila)
8.52	*GABRQ*	55879	gamma-aminobutyric acid receptor
8.37	*LEF1*	51176	lymphoid enhancer-binding factor 1, transcript variant 1
8.20	*KIF24*	347240	kinesin family member 24
7.69	*MPZL3*	196264	myelin protein zero-like 3
7.62	*FAM155A*	728215	family with sequence similarity 155, member A
7.20	*MYO15B*	80022	myosin XVB pseudogene
7.14	*TMTC2*	160335	transmembrane and tetratricopeptide repeat containing 2
6.99	*GLYATL1*	92292	glycine-N-acyltransferase-like 1
6.50	*DNAH11*	8701	dynein, axonemal, heavy chain 11
6.46	*GPRC5C*	55890	G protein-coupled receptor, family C, group 5, member C, transcript variant 1
6.00	*CDCA7L*	55536	cell division cycle associated 7-like, transcript variant 1
5.69	*EEF1D*	1936	eukaryotic translation elongation factor 1 delta
5.42	*HMGN1*	3150	high mobility group nucleosome binding domain 1
5.38	*FOXF1*	2294	forkhead box F1
5.26	*PDE7A*	5150	phosphodiesterase 7A, transcript variant 2
5.23	*DQX1*	165545	DEAQ box RNA-dependent ATPase 1
5.18	*EDNRB*	1910	endothelin receptor type B, transcript variant 2
5.05	*AMY1C*	278	amylase, alpha 1C (salivary)

Supplementary Table S2. Primers used in this study

Real-time PCR Primer	Sequence (5' to 3')
DLX4 (S)	TAA ACC AGC GTT TCC AGC AC
DLX4 (AS)	CTT ATA CTT GGA GCG TTT GTT CTG A
NANOG (S)	TCC AAC ATC CTG AAC CTC AGC TA
NANOG (AS)	AGT CGG GTT CAC CAG GCA TC
REX1 (S)	GTC AAA TAA CCT GAA AGC CCA CAT C
REX1 (AS)	AAT CAA TGA GGC ATG TTT GTC ACT G
OCT3/4 (End-S)	GAC AGG GGG AGG GGA GGA GCT AGG
OCT3/4 (End-AS)	CTT CCC TCC AAC CAG TTG CCC CAA AC
c-MYC (S)	CGG ATT CTC TGC TCT CCT CGA C
c-MYC (AS)	CCT CCA GCA GAA GGT GAT CCA
CDKN1A (p21) (S)	GGT GGC AGT AGA GGC TAT GGA CA
CDKN1A (p21) (AS)	GGC TCA ACG TTA GTG CCA GGA
CDKN2A (p16) (S)	GGC ACC AGA GGC AGT AAC CA
CDKN2A (p16) (AS)	GGA CCT TCG GTG ACT GAT GAT CTA A
CDKN2B (p15) (S)	GGC AGA CAG GTT TAG CTG TTT CAT C
CDKN2B (p15) (AS)	CCA CAA TGG AGC TAG AAG CAG GA
CDH1 (S)	AAG TGC TGC AGC CAA AGA CAG A
CDH1 (AS)	AAA TTG CCA GGC TCA ATG ACA AG
GAPDH (S)	GCA CCG TCA AGG CTG AGA AC
GAPDH (AS)	TGG TGA AGA CGC CAG TGG A
**Genomic PCR Primer**	**Sequence (5' to 3')**
OCT3/4 (Tg-S)	CCC CAG GGC CCC ATT TTG GTA CC
SOX2 (Tg-S)	GGC ACC CCT GGC ATG GCT CTT GGC TC
KLF4 (Tg-S)	ACG ATC GTG GCC CCG GAA AAG GAC C
c-MYC (Tg-S)	CAA CAA CCG AAA ATG CAC CAG CCC CAG
pMXs-AS	TTA TCG TCG ACC ACT GTG CTG CTG

Abbreviations: S, sense primers; AS, antisense primers; End-S, sense primers specifically for endogenous genes; End-AS, antisense primers specifically for endogenous genes; Tg-S, sense primers specifically for transgenes; pMXs-AS, antisense primer for all transgenes (*OCT3/4*, *SOX2*, *KLF4*, *c-MYC*).

Figure S1.

a

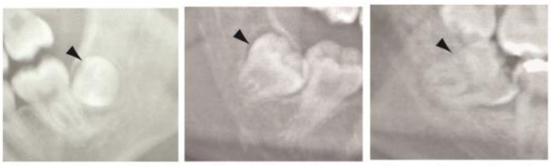

b

DPC lines used in this study

Cell lines	Sex	Age	Stage	Reprogramming efficiency assay	DNA microarray	Analysis of DLX4 expressioı
DP1	M	14	CC	√	√	√
DP28	M	14	CC		√	√
DP31	F	14	CC	√		√
DP49	F	12	CC			√
DP75	M	24	RC	√	√	√
DP81	M	16	RF		√	
DP87	F	20	RF	√		√
DP94	F	16	RF	√		√
DP101	M	16	RF		√	
DP133	M	27	RC	√	√	√
DP159	M	23	RC		√	√
DP165	F	24	RC	√		√
DP166	F	23	RC	√		√
DP193	M	60	RC	√	√	√

Figure S2.

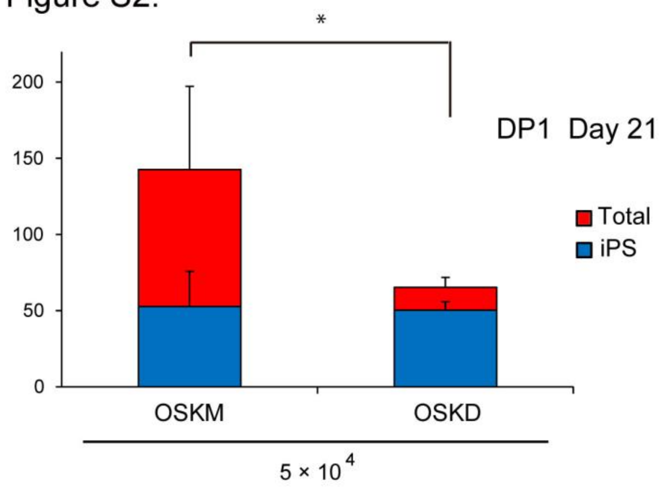

Figure S3.

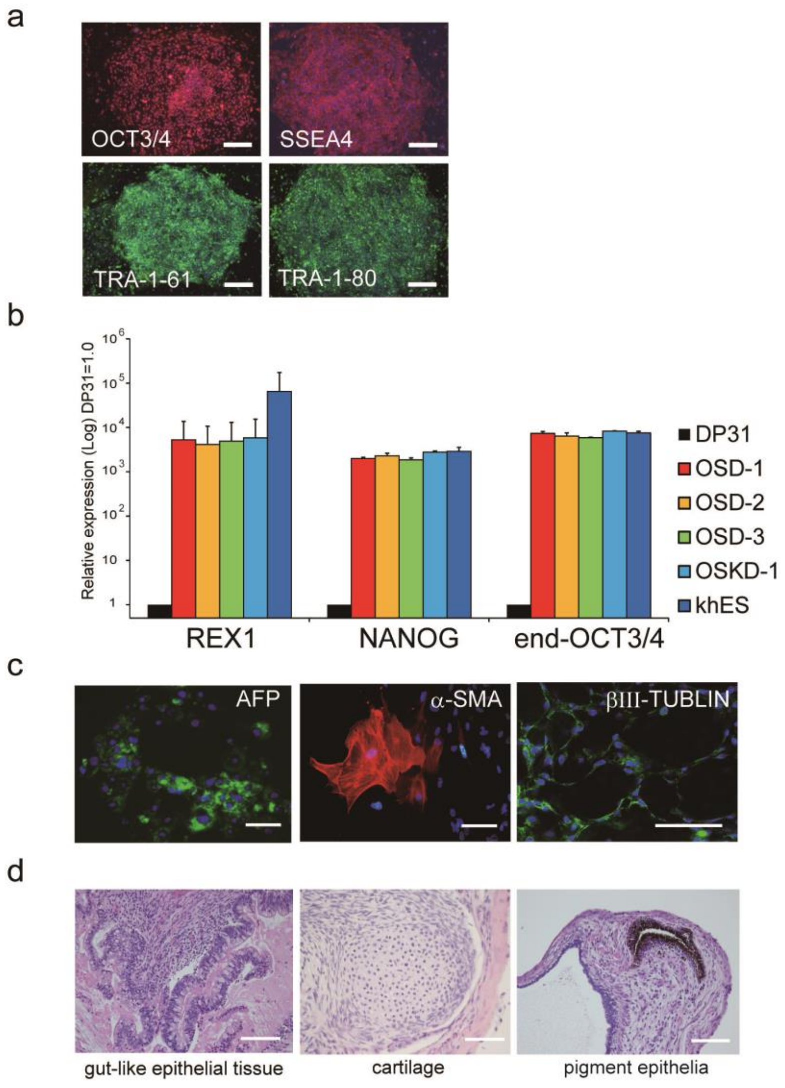

a
OCT3/4
SSEA4
TRA-1-61
TRA-1-80
b
Relative expression (Log) DP31=1.0
10^6
10^5
10^4
10^3
10^2
10
1
REX1
NANOG
end-OCT3/4
DP31
OSD-1
OSD-2
OSD-3
OSKD-1
khES
c
AFP
α-SMA
βIII-TUBLIN
d
gut-like epithelial tissue
cartilage
pigment epithelia

Figure S4.

a

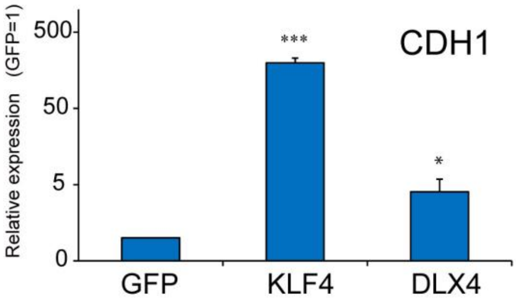

CDH1
Relative expression (GFP=1)
500
50
5
0
***
*
GFP
KLF4
DLX4

b

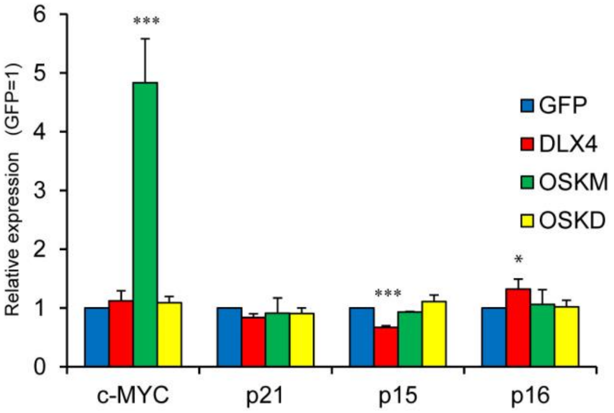

Relative expression (GFP=1)
6
5
4
3
2
1
0
***
***
*
GFP
DLX4
OSKM
OSKD
c-MYC
p21
p15
p16
